# Postoperative pain of patients with necrotic teeth with apical periodontitis following single visit endodontic treatment versus multiple visit endodontic treatment using triple antibiotic paste: a randomized clinical trial

**DOI:** 10.12688/f1000research.19936.1

**Published:** 2019-07-26

**Authors:** Safeya AbdurRahman, Saied M. Abdel Aziz, Shaimaa I. Gawdat, Ahmed M. AbdalSamad

**Affiliations:** 1 Department of Endodontics, Cairo University, Cairo, Egypt; 2 Department of Oral and Maxillofacial Radiology, Cairo University, Cairo, Egypt

**Keywords:** Apical periodontitis, multiple visits, triple antibiotic paste, interappointment dressing

## Abstract

**Background:** A randomized clinical trial was conducted to compare the postoperative pain following endodontic treatment of necrotic teeth with apical periodontitis. Treatments were performed in multiple visits with application of triple antibiotic paste interappointment dressing or single visit without interappointment dressing.

**Methods:** In total 44 participants were assigned randomly into two groups. Group A: multiple visit endodontic treatment with triple antibiotic paste interappointment dressing; group B: single visit endodontic treatment without interappointment dressing. Postoperative pain of participants was assessed after 24, 48, 72 hours and one week using numerical rating scale.

**Results:** No statistically significant difference was found in postoperative pain after 24, 48, 72 hours and one week between the two groups.

**Conclusion: **Triple antibiotic paste as an interappointment dressing in multiple visits endodontic treatment was not proved to reduce the postoperative pain compared to a single visit in patients with necrotic teeth with apical periodontitis who did not have an interappointment dressing.

**Trial registration:** clinicaltrials.gov, NCT02947763. Date: 28th October 2016.

## Introduction

Apical periodontitis (AP) arises primarily by continuous bacterial irritation from the root canals ^1^. AP significantly reduces the endodontic success rate ^2^. Therefore, treatment of AP should aim to completely eliminate the underlying root canal infection ^3^, either by chemomechanical preparation or placement of an interappointment dressing ^4^.

Recent systematic reviews found that multiple visits with calcium hydroxide did not improve postoperative pain and flare-up and radiographic healing compared to single visit endodontic treatment without interappointment dressing ^5– 8^. Consequently, the search for a better antimicrobial alternative is required ^9^. Previously, triple antibiotic paste (TAP) has been shown to effectively reduce the bacterial load in necrotic teeth ^10^.

The aim of the present study was to compare the postoperative pain following endodontic treatment, of necrotic teeth having apical periodontitis, either performed in single visit or in multiple visits with application of triple antibiotic paste interappointment dressing.

## Methods

### Trial design

The study was registered on *clinical trials.gov* and the registration number is NCT02947763.

The protocol was approved by the Committee of Ethics, Faculty of Dentistry, Cairo University, Egypt (approval no 16562). Participants were asked to sign a printed informed consent that explained the study aim, alternative treatments to endodontic treatment, and the investigator’s instructions.

The trial design of this study was a parallel, randomized, clinical design with allocation ratio 1:1. This article follows the CONSORT 2010 statement and a copy of the CONSORT checklist can be found in the Data availability section.

The study began in November 2016 and was completed in February 2018.

### Sample size calculation

Prior data ^11^ indicated that standard deviation of pain score was 20.3. If the true difference in the intervention and control is 20.6, we should study 16 in each group to be able to reject the null hypothesis that the population means of the intervention and control groups are equal with probability (power) 0.8. The Type I error probability associated with this test of this null hypothesis was 0.05. The size of the sample was increased to 22 per group to correct for non-parametric usage and to substitute for any drop-out.

### Participants

In total, 44 adults, medically-free with an age range of 16–55 years were selected for the study. All had a necrotic tooth with a periapical lesion confirmed radiographically (minimum size 2 × 2 mm). The participants were enrolled by SA.

Exclusion criteria included teeth previously accessed or endodontically treated; vital or necrotic teeth without periapical lesion; patients allergic to metronidazole, ciprofloxacin, or doxycycline or those with significant medical conditions; patients who took analgesic tablets before treatment up to 12 hours previously; and pregnant women.

### Treatment procedures

The teeth were tested with an electric pulp test (Denjoy DY310 Dental Pulp Tester; Denjoy, Henan, China) to determine pulp sensitivity. Radiographs were taken using a photo stimulable phosphor plate wireless sensor (SOREDEX, DIGORA) to detect periapical lesions.

Preoperative pain was recorded using a numerical rating scale (NRS) where 0 indicates no pain and 10 indicates pain as terrible as it could be ^12^. Pain intensity was categorized into either: none (0); mild (1–3); moderate (4–6); and severe (7–10) ^13^.

Local anesthesia was administered if needed (Ubistesin™ Articaine HCl 4% & Adrenaline 1:100,000 3M Australia). Isolation of the tooth with rubber dam and preparation of access cavity was performed and the root canal was instrumented by hybrid technique. Coronal shaping was performed with Gates-Glidden drills (MANI, Japan) sizes 4, 3 and 2. Working length was measured using apex locator (J Morita USA) and ascertained using radiograph, where it was set 1 mm away from the radiographic apex. The apical part was instrumented using stainless steel K-files (MANI, Japan); the master apical file size was set 3–4 sizes larger than the initial file. The middle part was instrumented using 3–5 stainless steel H-files.

Irrigation was done using sodium hypochlorite 2.6% (Clorox®, Egypt), using plastic disposable syringe with needle gauge 27, between successive instruments. Lubrication was done using EDTA gel (QMETA, Korea). Final irrigation was done with 5 ml 17% EDTA solution to remove the smear layer (17% EDTA solution, Prevest DenPro Limited, India). The final wash was done using saline. Master cone-fit radiograph was taken to ensure proper length and preparation. The canals were dried with paper points.

### Randomization

At this step, the participants were divided randomly into two groups with a table of random numbers from 1 to 44 generated by SMA using a freely available computer program with n=22/group. The allocation table was kept with an investigator not involved with participant enrollment (SIG). Numbers from 1 to 44 were written on 44 pieces of paper folded eight-times. Each paper was placed separately in a closed opaque envelope. Each participant was asked to pick one of the envelopes and the participant was assigned to the groups based on the number in the envelope.

Group A: multiple (two) visit endodontic treatment with triple antibiotic paste interappointment dressing; group B: single visit endodontic treatment without interappointment dressing.

For group A, triple antibiotic paste (ciprofloxacin, metronidazole, and doxycycline mixed with saline) was prepared and 1 mL of the mixed paste was placed into the canals with a 20-gauge needle of sterile plastic syringe. A sterile cotton pellet was placed, and glass ionomer was placed (Riva Self Cure, Australia). Preparation of the triple antibiotic paste followed the protocol of a previous study ^14^, using ciprofloxacin 250 mg tablets (EPICO, Egypt), metronidazole 500 mg tablets (Aventis, Egypt) and doxycycline 100 mg capsules (Pfizer, Egypt). The powder content of doxycycline capsule was placed in a sterile mortar. In the same mortar, a tablet of metronidazole and a tablet of ciprofloxacin were crushed and all are mixed with saline to a creamy paste. A second appointment was scheduled after at least 7 days. Under rubber dam isolation, the interappointment dressing was removed by H-files and 2.6% sodium hypochlorite and 17% EDTA irrigation followed by saline final wash. Then, obturation was performed using cold lateral condensation technique with resin sealer (ADSEAL, META, Korea). After obturation, glass ionomer was placed to seal the access cavity till final restoration.

For group B, no interappointment dressing was applied and endodontic treatment was ended in the same visit without placement of interappointment dressing. Obturation and sealing of access cavity were performed as in group A.

### Outcomes

***Primary outcomes.*** Postoperative pain at 24, 48, 72 hours and one week after instrumentation (first visit of Group A and the single visit of Group B); recorded by the participants using NRS in a pain diary.

***Secondary outcomes.*** Incidence of analgesic intake and number of tablets consumed in case of presence of moderate or severe postoperative pain. Participants were instructed to take one tablet of Ibuprofen 400 mg (NOVARITIS, Canada) every 6 hours and to report the number of tablets consumed.

### Blinding

The operator was blinded until the end of instrumentation until she saw the number of the envelope, then the operator either administered the interappointment dressing in group A or ended the endodontic treatment in a single visit (group B). Blinding of the operator to the end of treatment was difficult as there was only a single operator (SA). The participant did not know whether endodontic treatment was done in multiple or single visit; as another appointment was given to all participants whether to complete the endodontic treatment in Group A or for follow-up in Group B. The data analyst was blinded to the group assignment.

### Statistical analysis

All the data was collected and tabulated. Statistical analysis was performed by Microsoft Office 2013 (Excel) and statistical package SPSS version 22. The significance level was set at p-value ≤ 0.05. Non-parametric data was summarized as minimum, maximum and median. Chi-squared test was used to compare the incidence of studied parameters and Mann-Whitney test for analysis of severity of pain ^15^.

## Results

**Figure 1.**
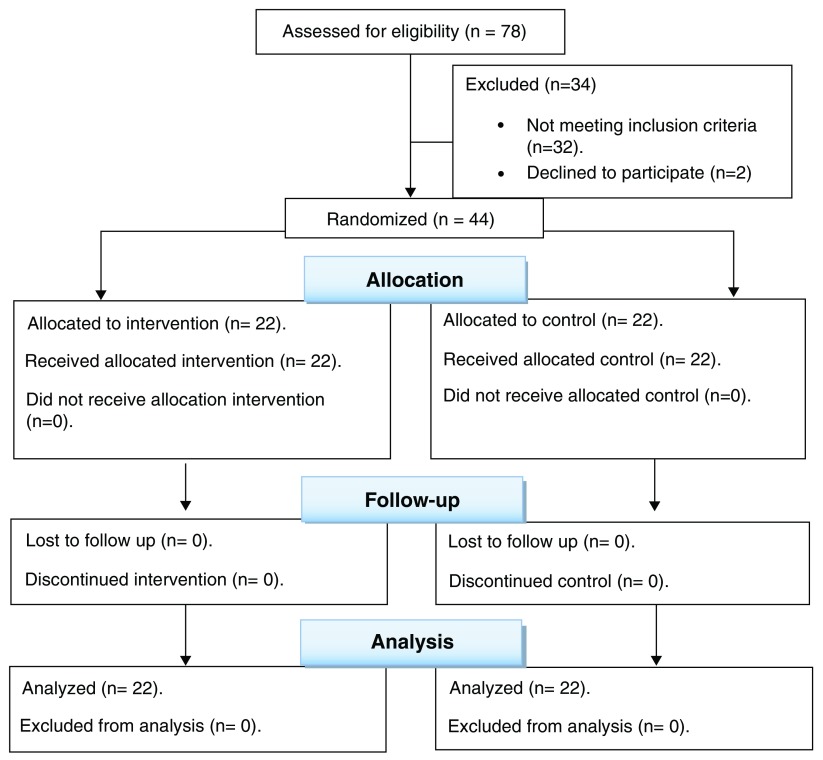
Consort 2010 flow diagram of the study design.

After enrollment of 78 patients, only 44 participants were included ( Figure 1). The age, gender, and preoperative pain did not differ significantly between the two groups; all participants in the two groups had preoperative no-to-mild pain ( Table 1).

**Table 1.** Demographic data and preoperative pain of the two groups.

	Group A * (n=22)	Group B ^♣^ (n=22)	*p*-value
**Age (years)** mean ± SD	31.27±13.5	37.9±10.3	0.07
**Gender [n (%)]** Women Men	18 (81.8%) 4 (18.2%)	17 (77.2%) 5 (22.8%)	0.7
**Preoperative pain (NRS)** Median Range	0 0–3	0 0–3	0.165
**Preoperative pain incidence [n (%)]** No pain Mild	18 (81.4%) 4 (18.6%)	14 (63.6%) 8 (36.4%)	0.175

*Multiple visits group; ^♣^ Single visit group; NRS = numerical rating scale; SD= standard deviation.

The data of the postoperative pain are shown in Table 2. There was no statistically significant difference between the two tested groups, either in the intensity nor the incidence of different pain categories. In both groups, there was a significant decrease in the incidence of pain at different follow-up periods ( *p* < 0.05; Table 3).

**Table 2.** Incidence and intensity of postoperative pain of the two groups.

Pain	Group A * (n=22)	Group B ^♣^ (n=22)	*p*-value
**After 24 hours**			
**Intensity (NRS)** Median Range	0 0–9	0 0–10	0.878
**Incidence [n (%)]** No pain Mild Moderate Severe	15 (68.2%) 3 (13.6%) 0 (0%) 4 (18.2%)	14 (63.6%) 5 (22.7%) 2 (9.1%) 1 (4.5%)	0.228
**After 48 hours**			
**Intensity (NRS)** Median Range	0 0–8	0 0–7	0.452
**Incidence [n (%)]** No pain Mild Moderate Severe	16 (72.7%) 2 (9.1%) 3 (13.6%) 1 (4.5%)	18 (81.8%) 2 (9.1%) 1 (4.5%) 1 (4.5%)	0.773
**After 72 hours**			
**Intensity (NRS)** Median Range	0 0–4	0 0–3	0.962
**Incidence [n (%)]** No pain Mild Moderate Severe	20 (90.9%) 1 (4.5%) 1 (4.5%) 0 (0%)	20 (90.9%) 2 (9.1%) 0 (0%) 0 (0%)	0.513
**After one week**			
**Intensity (NRS)** Median Range	0 0–0	0 0–0	1
**Incidence [n (%)]** No pain Mild Moderate Severe	22 (100%) 0 (0%) 0 (0%) 0 (0%)	22 (100%) 0 (0%) 0 (0%) 0 (0%)	1

*Multiple visits group; ^♣^ Single visit group. NRS=numerical rating score.

**Table 3.** Incidence of pain at different time intervals for each group.

	Preoperative	24hrs	48hrs	72hrs	1 week	*p-*value
**Group A** * **n** **%**	4 (18.18)	7 (31.8)	6 (27.2)	2 (9.1)	0 (0)	0.032
**Group B ^♣^** **n** **%**	8 (36.3)	8 (36.3)	4 (18.18)	2 (9.1)	0 (0)	0.005

* Multiple visits group; ^♣^ Single visit group.

There was no statistically significant difference between the two tested groups regarding the incidence of analgesic intake and number of analgesics tablets taken by the participants ( *p* > 0.05; Table 4).

**Table 4.** Incidence of analgesic intake and number of analgesics tablets of the two groups.

	Group A * n=22	Group B ^♣^ n=22	*p*-value
**Incidence of analgesic intake**			
**n** **%**	4 18.1	3 13.6	0.68
**No of tablets**			
**Median** **Minimum** **Maximum** **Total**	0 0 2 7	0 0 4 7	0.74 1

*Multiple visits group; ^♣^ Single visit group.

## Discussion

Presence of AP decreases the endodontic success rate, regarding postoperative pain and radiographic healing, by about 10%–15% ^2, 16^. Maximum removal of the bacteria and irritants causing AP is essential to achieve better prognosis ^17^. Placement of inter-appointment dressing has been previously recommended to completely disinfect the root canal system ^4^.

TAP has been found to remain active for 30 days ^18^ and shows better antibacterial efficacy than calcium hydroxide in previous *in-vitro* studies ^18– 28^. TAP has been used clinically in case reports and series to treat cases with large periapical lesions, when the use of calcium hydroxide cannot eliminate the symptoms ^29– 32^. Moreover, previous randomized clinical trials found that TAP is better than calcium hydroxide, as an intracanal medicament, both clinically and radiographically ^33– 35^.

In the present study, the intensity and the incidence at different pain categories did not differ statistically to a significant level (age, gender and preoperative pain were similarly distributed among both groups). This finding is in accordance to the results of previous studies comparing single visit versus multiple visits with calcium hydroxide ^36– 41^. During a previous literature search, the authors found no similar studies comparing single visit versus multiple visits with TAP.

In our study, the median postoperative pain of multiple visits group (with TAP) at all follow-up periods was 0 and the incidence of moderate and severe pain ranged from 18.2% to 0%. These results are comparable to the results of previous studies. Pai *et al*. ^35^ found no interappointment flare-up in the group treated with TAP after 1, 2, 3, 7, and 14 days (in diabetic patients). Prasad *et al*. ^33^ found that the mean of postoperative pain after one week was 0.86, while Bilgi *et al*. ^42^ found that the mean of postoperative pain after 24 and 48 hours was 0.08. 

In the present study, nearly 95% of the participants were asymptomatic after 72 hours postoperatively. Previous studies found that severe postoperative pain is reduced to a mild pain during this period of time ^43, 44^.

Within the conditions of this study, it could be concluded that postoperative pain was similar after performing endodontic treatment in multiple visits with triple antibiotic paste interappointment dressing or in a single visit.

## Data availability

### Underlying data

Figshare: Postoperative Pain after Single versus Multiple Visits Endodontic Treatment of Necrotic Teeth with Apical Periodontitis with Triple Antibiotic Paste: A Randomized Clinical Trial. Dataset demographic data and postoperative pain, https://doi.org/10.6084/m9.figshare.8797592.v1 ^45^

### Reporting guidelines

Figshare: CONSORT checklist, https://doi.org/10.6084/m9.figshare.8797592.v1 ^45^

Data are available under the terms of the Creative Commons Zero "No rights reserved" data waiver (CC0 1.0 Public domain dedication).

## References

[1] Katebzadeh N Sigurdsson A Trope M: Radiographic evaluation of periapical healing after obturation of infected root canals: an *in vivo* study. *Int Endod J.* 2000;33(1):60–6. 10.1046/j.1365-2591.2000.00301.x 11307475

[2] Friedman S Abitbol S Lawrence HP: Treatment outcome in endodontics: the Toronto Study. Phase 1: initial treatment. *J Endod.* 2003;29(12):787–93. 10.1097/00004770-200312000-00001 14686806

[3] Tervit C Paquette L Torneck CD: Proportion of healed teeth with apical periodontitis medicated with two percent chlorhexidine gluconate liquid: a case-series study. *J Endod.* 2009;35(9):1182–5. 10.1016/j.joen.2009.05.010 19720213

[4] Law A Messer H: An evidence-based analysis of the antibacterial effectiveness of intracanal medicaments *J Endod.* 2004;30(10):689–94. 10.1097/01.DON.0000129959.20011.EE 15448460

[5] Anjaneyulu K Nivedhitha MS: Influence of calcium hydroxide on the post-treatment pain in Endodontics: A systematic review. *J Conserv Dent.* 2014;17(3):200–7. 10.4103/0972-0707.131775 24944439 PMC4056387

[6] Wong AW Zhang C Chu CH: A systematic review of nonsurgical single-visit versus multiple-visit endodontic treatment. *Clin Cosmet Investig Dent.* 2014;6:45–56. 10.2147/CCIDE.S61487 24855389 PMC4020891

[7] Figini L Lodi G Gorni F: Single versus multiple visits for endodontic treatment of permanent teeth: a Cochrane systematic review. *J Endod.* 2008;34(9):1041–7. 10.1016/j.joen.2008.06.009 18718362

[8] Su Y Wang C Ye L: Healing rate and post-obturation pain of single- versus multiple-visit endodontic treatment for infected root canals: a systematic review. *J Endod.* 2011;37(2):125–32. 10.1016/j.joen.2010.09.005 21238790

[9] Waltimo T Trope M Haapasalo M: Clinical efficacy of treatment procedures in endodontic infection control and one year follow-up of periapical healing. *J Endod.* 2005;31(12):863–6. 10.1097/01.don.0000164856.27920.85 16306819

[10] Mohammadi Z Abbott PV: On the local applications of antibiotics and antibiotic-based agents in endodontics and dental traumatology. *Int Endod J.* 2009;42(7):555–67. 10.1111/j.1365-2591.2009.01564.x 19467048

[11] Patil AA Joshi SB Bhagwat SV: Incidence of postoperative pain after single visit and two visit root canal therapy: a randomized controlled trial. *J Clin Diagn Res.* 2016;10(5):ZC09–12. 10.7860/JCDR/2016/16465.7724 27437339 PMC4948515

[12] Williamson A Hoggart B: Pain: a review of three commonly used pain rating scales. *J Clin Nurs.* 2005;14(7):798–804. 10.1111/j.1365-2702.2005.01121.x 16000093

[13] Jalalzadeh SM Mamavi A Shahriari S: Effect of pretreatment prednisolone on postendodontic pain: a double-blind parallel-randomized clinical trial. *J Endod.* 2010;36(6):978–81. 10.1016/j.joen.2010.03.015 20478449

[14] Estefan BS El Batouty KM Nagy MM: Influence of Age and Apical Diameter on the Success of Endodontic Regeneration Procedures. *J Endod.* 2016;42(11):1620–5. 10.1016/j.joen.2016.06.020 27623497

[15] Chan YH: Biostatistics 102: quantitative data--parametric & non-parametric tests. *Singapore Med J.* 2003;44(8):391–6. 14700417

[16] Marquis VL Dao T Farzaneh M: Treatment outcome in endodontics: the Toronto Study. Phase III: initial treatment. *J Endod.* 2006;32(4):299–306. 10.1016/j.joen.2005.10.050 16554199

[17] Nixdorf DR Moana-Filho EJ Law AS: Frequency of persistent tooth pain after root canal therapy: a systematic review and meta-analysis. *J Endod.* 2010;36(2):224–230. 10.1016/j.joen.2009.11.007 20113779 PMC2832800

[18] Maniglia-Ferreira C de Almeida-Gomes F Pinto MM: *In vitro* evaluation of the antimicrobial effects of different intracanal medications in necrotic immature teeth. *Eur Arch Paediatr Dent.* 2016;17(4):251–5. 10.1007/s40368-016-0236-x 27412439

[19] Adl A Shojaee NS Motamedifar M: A comparison between the antimicrobial effects of triple antibiotic paste and calcium hydroxide against entrococcus faecalis. *Iran Endod J.* 2012;7(3):149–55. 23056135 PMC3467137

[20] Sabrah AH Yassen GH Gregory RL: Effectiveness of antibiotic medicaments against biofilm formation of *enterococcus faecalis* and porphyromonas gingivalis. *J Endod.* 2013;39(11):1385–9. 10.1016/j.joen.2013.05.003 24139259

[21] Ordinola-Zapata R Bramante CM Minotti PG: Antimicrobial activity of triantibiotic paste, 2% chlorhexidine gel, and calcium hydroxide on an intraoral-infected dentin biofilm model. *J Endod.* 2013;39(1):115–8. 10.1016/j.joen.2012.10.004 23228269

[22] Shokraneh A Farhad AR Farhadi N: Antibacterial effect of triantibiotic mixture versus calcium hydroxide in combination with active agents against *Enterococcus faecalis* biofilm. *Dent Mater J.* 2014;33(6):733–8. 10.4012/dmj.2014-090 25297855

[23] Adl A Hamedi S Sedigh Shams M: The ability of triple antibiotic paste and calcium hydroxide in disinfection of dentinal tubules. *Iran Endod J.* 2014;9(2):123–6. 24688581 PMC3961590

[24] Mozayeni MA Haeri A Dianat O: Antimicrobial effects of four intracanal medicaments on *enterococcus faecalis*: an *In vitro* study. *Iran Endod J.* 2014;9(3):195–8. 25031593 PMC4099951

[25] Devaraj S Jagannathan N Neelakantan P: Antibiofilm efficacy of photoactivated curcumin, triple and double antibiotic paste, 2% chlorhexidine and calcium hydroxide against Enterococcus fecalis *In vitro*. *Sci Rep.* 2016;6:24797. 10.1038/srep24797 27097667 PMC4838845

[26] Lakhani AA Sekhar KS Gupta P: Efficacy of Triple Antibiotic Paste, Moxifloxacin, Calcium Hydroxide and 2% Chlorhexidine Gel In Elimination of *E. Faecalis*: An *In vitro* Study. *J Clin Diagnostic Res.* 2017;11(1):ZC06–ZC09. 10.7860/JCDR/2017/22394.9132 28274034 PMC5324479

[27] Mehta S Verma P Tikku AP: Comparative evaluation of antimicrobial efficacy of triple antibiotic paste, calcium hydroxide, and a proton pump inhibitor against resistant root canal pathogens. *Eur J Dent.* 2017;11(1):53–7. 10.4103/ejd.ejd_159_16 28435366 PMC5379835

[28] Abbaszadegan A Dadolahi S Gholami A: Antimicrobial and Cytotoxic Activity of *Cinnamomum zeylanicum*, Calcium Hydroxide, and Triple Antibiotic Paste as Root Canal Dressing Materials. *J Contemp Dent Pr.* 2018;17(2):105–13. 10.5005/jp-journals-10024-1811 27206997

[29] Er K Kuştarci A Ozan U: Nonsurgical endodontic treatment of dens invaginatus in a mandibular premolar with large periradicular lesion: a case report. *J Endod.* 2007;33(3):322–4. 10.1016/j.joen.2006.09.001 17320725

[30] Taneja S Kumari M Parkash H: Nonsurgical healing of large periradicular lesions using a triple antibiotic paste: A case series. *Contemp Clin Dent.* 2010;1(1):31–5. 10.4103/0976-237X.62519 22114375 PMC3220065

[31] Taneja S Kumari M: Use of triple antibiotic paste in the treatment of large periradicular lesions. *J Investig Clin Dent.* 2012;3(1):72–6. 10.1111/j.2041-1626.2011.00082.x 22298525

[32] Dhillon JS Amita Saini SK: Healing of a large periapical lesion using triple antibiotic paste and intracanal aspiration in nonsurgical endodontic retreatment. *Indian J Dent.* 2014;5(3):161–5. 10.4103/0975-962X.140843 25565747 PMC4213876

[33] Prasad LK Tanwar BS Kumar KN: Comparison of calcium hydroxide and triple antibiotic paste as intracanal medicament in emergency pain reduction: *in vivo* study. *Int J Care Res.* 2016;4:244–6. Reference Source

[34] Johns DA Varughese JM Thomas K: Clinical and radiographical evaluation of the healing of large periapical lesions using triple antibiotic paste, photo activated disinfection and calcium hydroxide when used as root canal disinfectant. *J Clin Exp Dent.* 2014;6(3):e230–6. 10.4317/jced.51324 25136422 PMC4134850

[35] Pai S Vivekananda Pai AR Thomas MS: Effect of calcium hydroxide and triple antibiotic paste as intracanal medicaments on the incidence of inter-appointment flare-up in diabetic patients: An *in vivo* study. *J Conserv Dent.* 2014;17(3):208–11. 10.4103/0972-0707.131776 24944440 PMC4056388

[36] El Mubarak AH Abu-bakr NH Ibrahim YE: Postoperative pain in multiple-visit and single-visit root canal treatment. *J Endod.* 2010;36(1):36–9. 10.1016/j.joen.2009.09.003 20003932

[37] Ghoddusi J Javidi M Zarrabi MH: Flare-ups incidence and severity after using calcium hydroxide as intracanal dressing. *N Y State Dent J.* 2006;72(4):24–8. 16925009

[38] Sathorn C Parashos P Messer H: The prevalence of postoperative pain and flare-up in single- and multiple-visit endodontic treatment: a systematic review. *Int Endod J.* 2008;41(2):91–99. 10.1111/j.1365-2591.2007.01316.x 17956561

[39] Ince B Ercan E Dalli M: Incidence of postoperative pain after single- and multi-visit endodontic treatment in teeth with vital and non-vital pulp. *Eur J Dent.* 2009;3(4):273–9. 19826598 PMC2761157

[40] Akbar I Iqbal A Al-Omiri MK: Flare-up rate in molars with periapical radiolucency in one-visit vs two-visit endodontic treatment. *J Contemp Dent Pract.* 2013;14(3):414–8. 10.5005/jp-journals-10024-1337 24171982

[41] Tarale K: Post-operative Pain Analysis between Single Visit and Two Visit Root Canal Treatments using Visual Analogue Scale: An *in vivo* Study. *J Dent Allied Sci.* 2013;2(1):8–15. 10.4103/2277-4696.159256

[42] Bilgi PS Shah NC Mehta J: Comparative evaluation of mixture of calcium hydroxide and chlorhexidine, with triple antibiotic paste and combination of calcium hydroxide, chlorhexidine, and lycopene on incidence of interappointment flare-up: an *in vivo* study. *Int J Clin Dent Res.* 2017;1(1):10–4. Reference Source

[43] Genet JM Wesselink PR Thoden van Velzen SK: The incidence of preoperative and postoperative pain in endodontic therapy. *Int Endod J.* 1986;19(5):221–229. 10.1111/j.1365-2591.1986.tb00482.x 3473042

[44] Pak JG White SN: Pain prevalence and severity before, during, and after root canal treatment: a systematic review. *J Endod.* 2011;37(4):429–38. 10.1016/j.joen.2010.12.016 21419285

[45] Abdurrahman S: Postoperative Pain after Single versus Multiple Visits Endodontic Treatment of Necrotic Teeth with Apical Periodontitis with Triple Antibiotic Paste: A Randomized Clinical Trial. *figshare. Dataset.* 2019 10.6084/m9.figshare.8797592.v1 PMC6993827 32047601

