# Supplementary material for: Postoperative pain of patients with necrotic teeth with apical periodontitis following single visit endodontic treatment versus multiple visit endodontic treatment using triple antibiotic paste: a randomized clinical trial
Source: F1000Res. 2019 Jul 26;8:1203. [Version 1] doi: 10.12688/f1000research.19936.1 (PMC6993827; doi:10.12688/f1000research.19936.1)
Supplement: Peer review history

# Peer review: Reviewer response for version 1

**DOI:** 10.5256/f1000research.21880.r51772

**Published:** 2020-01-27

**Authors:** A. R. Vivekananda Pai, Htoo Htoo Kyaw Soe

**Affiliations:** 1 Department of Conservative Dentistry and Endodontics, Faculty of Dentistry, Melaka-Manipal Medical College (Manipal Academy of Higher Education [MAHE]), Malacca City, Malaysia; 2 Department of Community Medicine, Melaka-Manipal Medical College (Manipal Academy of Higher Education [MAHE]), Malacca City, Malaysia

**Abstract:**

**Background:**

 - Suggested grammatical/formatting corrections:

 1) Page no. 1, Paragraph no. 1, Sentence line no. 4:

- Stated as: “dressing or single visit without interappointment dressing.”
- Suggested correction: dressing or in single visit without an interappointment dressing.

**Conclusion:**

 - Since the number of visits is not the same/not standardized between intervention and control groups, and is a variable factor between them, the conclusion needs to be revised.

 - Suggested correction for the conclusion:

 1) Page no. 1, Paragraph no. 1, Sentence line no. 1-4:

- Stated as: “Triple antibiotic paste as an interappointment dressing in multiple visits endodontic treatment was not proved to reduce the postoperative pain compared to a single visit in patients with necrotic teeth with apical periodontitis who did not have an interappointment dressing.”
- Suggested correction: There is no significant difference in the postoperative pain when endodontic treatment is carried out in patients for necrotic teeth with apical periodontitis either in multiple visits with an interappointment dressing of triple antibiotic paste or in a single visit without an interappointment dressing.

**Introduction:**

 - It is clear and appropriate, but very brief. Therefore, it needs elaboration on the contents related to postoperative pain/flare-up, particularly following single visit vs. multiple visit endodontics, and triple antibiotic paste.  

**Methods:**

**Trial design:**

 - CONSORT guidelines have been followed and CONSORT checklist for reporting has been mentioned. 

 - Suggested grammatical/formatting corrections:

 1) Page no. 3, Paragraph no. 2, Sentence line no. 2:

- Stated as: “(approval no 16562).”
- Suggested correction: (approval no. 16562).

                                                            Or

                                                (approval number 16562).

**Sample size calculation:**

 - Sample size calculation is clear, but the authors must state the percentage of anticipated drop-out rate.

 - Suggested grammatical/formatting corrections:

 1) Page no. 3, Paragraph no. 1, Sentence line no. 7:

- Stated as: “size of the sample was…...”
- Suggested correction: size of the sample (n) was…...  

**Participants:**

 - Selection/eligibility criteria have been made explicit.

 - Suggested grammatical/formatting corrections:

 1) Page no. 3, Paragraph no. 1, Sentence line no. 4:

- Stated as: “The participants were enrolled by SA.”
- Suggested correction: The participants were enrolled by author SA.

**Treatment procedures:**

 - The proportion or ratio of antibiotics used for the TAP should be specified.

 - Suggested grammatical/formatting corrections:

 1) Page no. 3, Paragraph no. 1, Sentence line no. 1:

- Stated as: “electric pulp test”
- Suggested correction: electric pulp tester

**Randomization:**

 - Details of randomization and allocation concealment have been described.

 - Suggested grammatical/formatting corrections:

 1) Page no. 3, Paragraph no. 1, Sentence line no. 3:

- Stated as: “1 to 44 generated by SMA……...”
- Suggested correction: 1 to 44 generated by author SMA……...

2) Page no. 3, Paragraph no. 1, Sentence line no. 5:

- Stated as: “The allocation table was kept with an investigator not involved with participant enrollment (SIG).”
- Suggested correction: The allocation table was kept with an investigator not involved with participant enrollment (author SIG).

3) Page no. 4, Paragraph no. 3, Sentence line no. 5:

- Stated as: “glass ionomer was placed……….”
- Suggested correction: glass ionomer cement was placed……….

4) Page no. 4, Paragraph no. 3, Sentence line no. 12:

- Stated as: “and all are mixed……….”
- Suggested correction: and all were mixed……….

5) Page no. 4, Paragraph no. 3, Sentence line no. 19:

- Stated as: “glass ionomer was placed……….”
- Suggested correction: glass ionomer cement was placed……….

6) Page no. 4, Paragraph no. 4, Sentence line no. 2:

- Stated as: “endodontic treatment was ended……….”
- Suggested correction: endodontic treatment was completed……….

**Blinding:**

 - Procedure of blinding has been carried out. However, type of blinding is not specified.

 - Type of blinding should be stated.

 - Suggested grammatical/formatting corrections:

 1) Page no. 4, Paragraph no. 1, Sentence line no. 1-2:

- Stated as: “The operator was blinded until the end of instrumentation until she saw the number of the envelope,…….”
- Suggested correction: The operator was blinded until the end of instrumentation and saw the number in the envelope,…….

2) Page no. 4, Paragraph no. 1, Sentence line no. 3:

- Stated as: “or ended the endodontic treatment………”
- Suggested correction: or completed the endodontic treatment………

3) Page no. 4, Paragraph no. 1, Sentence line no. 5-7:

- Stated as: “Blinding of the operator to the end of treatment was difficult as there was only a single operator (SA). The participant did not know whether endodontic treatment was done in multiple or single visit;……”
- Suggested correction: Blinding of the operator until the end of treatment was difficult as there was only a single operator (author SA). The participants did not know whether endodontic treatment was done in multiple or in single visit;……  

**Statistical analysis:**

 - Appropriate statistical tests have been carried out.

 - Suggested grammatical/formatting corrections:

 1) Page no. 4, Paragraph no. 1, Sentence line no. 5:

- Stated as: “Chi-squared test was……”
- Suggested correction: Chi-square test was……

 

**Results:**

 - All the outcomes measured in the study have been included in the results section.

 - The name of the statistical test/tests applied for each table (i.e. Tables 1, 2, 3, and 4) should be stated as a foot note.

 - The median value of pain intensity, which has been recorded using numerical rating scale (NRS), should be described together with 1 ^st^ quartile (Q1) and 3 ^rd^ quartile (Q3).

*- p*-value 1 must be reported as 0.999 (Tables 2 and 4).

*- p*-values must be reported to 3 decimal places (Tables 1 and 4).

 - In table 4, the total number of tablets taken is shown as 7 under both the groups (Page no. 6, Table no. 4, Row no. 5), whereas the maximum number is 2 and 4 for groups A and B, respectively. This table should be checked to identify whether there is any error in reporting the values.  

 - Suggested grammatical/formatting corrections:

 1) Page no. 4, Paragraph no. 1, Sentence line no. 3-4:

- Stated as: “all participants in the two groups had preoperative no-to-mild pain (Table 1).”
- Suggested correction: all the participants in both the groups had preoperative no-to-mild pain (Table 1).

2) Page no. 4, Paragraph no. 2, Sentence line no. 1:

- Stated as: “The data of the postoperative pain are shown in Table 2.”
- Suggested correction: The data of the postoperative pain is shown in Table 2.

3) Page no. 4, Paragraph no. 2, Sentence line no. 3:

- Stated as: “either in the intensity nor the incidence of different pain categories.”
- Suggested correction: either in the intensity or in the incidence of different pain categories.

                                                   Or

            neither in the intensity nor in the incidence of different pain categories.

 4) Page no. 5, Figure 1, Sentence line no. 10:

- Stated as: “Did not receive allocation intervention (n=0).”
- Suggested correction: Did not receive allocated intervention (n=0).

5) Page no. 6, Table 3, Row no. 1:

- Stated as:                     24hrs    48hrs    72hrs    1                                                                                                                                               week
- Suggested correction:   24hrs.   48hrs.   72hrs.  1 week or 1 wk.           

6) Page no. 6, Table 3, Row no. 2-3:

- Stated as:                          Group A*                   Group B*

                                                         n%                              n%

- Suggested correction:    Group A* [n(%)]            Group B* [n(%)]

7) Page no. 6, Table 4, Row no. 1:

- Stated as:                            Group A*                 Group B*

                                                           n=22                        n=22

- Suggested correction:         Group A*                  Group B*

                                                          (n=22)                       (n=22)

 8) Page no. 6, Table 4, Row no. 3:

- Stated as:                              n                       4                  3

                                                         %                     18.1             13.6

- Suggested correction:          [n(%)]               4 (18.1%)     3 (13.6%)                                                                   

9) Page no. 6, Table 4, Row no. 4:

- Stated as: “No of tablets”                           
- Suggested correction: Number of tablets  

                                                          Or

                                                  No. of tablets

**Discussion:**

 - It needs elaboration with justification on the methodology, particularly emphasizing on the type of tooth selection and the use of hybrid technique of cleaning and shaping.

 - Similarly, it needs more explanation with justification on results.

 - Suggested grammatical/formatting corrections:

 1) Page no. 4, Paragraph no. 2, Sentence line no. 1:

- Stated as: “and shows better antibacterial efficacy than calcium hydroxide……...”
- Suggested correction: and has shown better antibacterial efficacy than calcium hydroxide……...

**Conclusion:**

- Stated as: “Within the conditions of this study, it could be concluded that postoperative pain was similar after performing endodontic treatment in multiple visits with triple antibiotic paste interappointment dressing or in a single visit.”
- Suggested correction: Within the limitations of this study, it could be concluded that there is no significant difference in the postoperative pain when endodontic treatment is carried out in patients for necrotic teeth with apical periodontitis either in multiple visits with an interappointment dressing of triple antibiotic paste or in a single visit without an interappointment dressing.

We confirm that we have read this submission and believe that we have an appropriate level of expertise to confirm that it is of an acceptable scientific standard, however we have significant reservations, as outlined above.

# Peer review: Reviewer response for version 1

**DOI:** 10.5256/f1000research.21880.r51777

**Published:** 2019-08-13

**Authors:** Reham Hassan

**Affiliations:** 1 Faculty of Dentistry, Minia University, Minya, Egypt

The current manuscript links between intra-canal medication, number of visits and the post-operative pain after endodontic treatment. While the results were insignificant, the antibiotic paste presents some disadvantages, like discoloration which was not reported.

 Overall, the study was well written and designed. Additionally, it fits in a Randomized Control Trial, which increases the significance of the research.

- **Introduction:** Which was well-written and covers the relevant theoretical data of literature.
- **Aim of the study:** Here the authors stated the objectives and goals of the study in an appropriate manner.
- **In the Methods**: The authors gave a detailed description of the employed Allocation concealment and sequence generation was described adequately. The concentration of the creamy antibiotic paste prepared was not mentioned.
- The discussion section fails to explain the results.

I confirm that I have read this submission and believe that I have an appropriate level of expertise to confirm that it is of an acceptable scientific standard.

# Peer review: Reviewer response for version 1

**DOI:** 10.5256/f1000research.21880.r51774

**Published:** 2019-08-12

**Authors:** Alireza Adl

**Affiliations:** 1 Department of Endodontics, Biomaterials Research Center, School of Dentistry, Shiraz University of Medical Sciences (SUMS), Shiraz, Iran

This article is aimed at comparing the postoperative pain following single or two-visit endodontic treatment of necrotic teeth with apical periodontitis. The subject has been previously investigated when calcium hydroxide was used as the intracanal medicament. The point of this article is using triple antibiotic paste as the inter-appointment medicament. The authors have shown that there is no difference between two groups of patients regarding post-operative pain. Although the subject is interesting, there are a few issues that need to be clarified:

1. The introduction is very short. I believe the authors should explain more about the post-operative pain following endodontic treatment, its mechanisms and incidence. Moreover they also should explain why and how triple antibiotic paste might be effective in reducing the post-operative pain.
2. In this study, only necrotic teeth with periapical lesions were included. These teeth are prone to flare up (a combination of pain and swelling). I am interested to know why the authors evaluated only pain and not swelling.
3. Normally in this kind of study only teeth with a single canal are included. However, in the methods part of this article, it is not clear what types of teeth were selected; single-rooted or multiple-rooted or both types? 

I confirm that I have read this submission and believe that I have an appropriate level of expertise to confirm that it is of an acceptable scientific standard, however I have significant reservations, as outlined above.
